# Rubber Hand Illusion under Delayed Visual Feedback

**DOI:** 10.1371/journal.pone.0006185

**Published:** 2009-07-09

**Authors:** Sotaro Shimada, Kensuke Fukuda, Kazuo Hiraki

**Affiliations:** 1 Department Electronics and Bioinformatics, School of Science and Technology, Meiji University, Tama-ku, Kawasaki, Japan; 2 Department General Systems Studies, The University of Tokyo, Meguro-ku, Tokyo, Japan; 3 Interfaculty Initiative in Information Studies, The University of Tokyo, Meguro-ku, Tokyo, Japan; University of Minnesota, United States of America

## Abstract

**Background:**

Rubber hand illusion (RHI) is a subject's illusion of the self-ownership of a rubber hand that was touched synchronously with their own hand. Although previous studies have confirmed that this illusion disappears when the rubber hand was touched asynchronously with the subject's hand, the minimum temporal discrepancy of these two events for attenuation of RHI has not been examined.

**Methodology/Principal Findings:**

In this study, various temporal discrepancies between visual and tactile stimulations were introduced by using a visual feedback delay experimental setup, and RHI effects in each temporal discrepancy condition were systematically tested. The results showed that subjects felt significantly greater RHI effects with temporal discrepancies of less than 300 ms compared with longer temporal discrepancies. The RHI effects on reaching performance (proprioceptive drift) showed similar conditional differences.

**Conclusions/Significance:**

Our results first demonstrated that a temporal discrepancy of less than 300 ms between visual stimulation of the rubber hand and tactile stimulation to the subject's own hand is preferable to induce strong sensation of RHI. We suggest that the time window of less than 300 ms is critical for multi-sensory integration processes constituting the self-body image.

## Introduction

The phenomenon called “rubber hand illusion (RHI)” has a critical impact on understanding how our brain organizes one's own body image that induces the sense of self-ownership [1]. RHI is the attribution of a rubber hand to one's own body and occurs when tactile stimulation to the invisible subject's hand and corresponding visual stimulation to the visible rubber hand are applied simultaneously. While it is still under debate how precisely the rubber hand should be spatially compatible or resemble the subject's hand to induce RHI [2]–[5], most studies found that RHI is greatly reduced when the tactile and visual stimulations are delivered asynchronously [1], [6]. These results indicate that temporal contiguity of tactile and visual stimulation is pivotal to RHI [2], [5].

However, the length of the temporal discrepancy between the tactile and visual stimulation for attenuation of RHI is unknown, as most studies used only two conditions regarding temporal contiguity: synchronous and asynchronous (or uncorrelated) conditions. Previous studies have suggested that a delay of approximately 500 ms is sufficient to reduce RHI [7], [8]. In one study [7], alternating brushstrokes were applied to the subject's hand and the rubber hand at 1 Hz in the asynchronous condition, which corresponds to a temporal discrepancy between the visual and tactile stimuli of 500 ms. In another study [8], temporal discrepancies between 500–1000 ms between the two stimuli was randomly assigned in the asynchronous condition. In both studies, RHI was greatly attenuated in the asynchronous condition. Thus a temporal discrepancy of more than 500 ms is considered to be sufficient to reduce RHI effects. Similarly, our previous near-infrared spectroscopy (NIRS) study [9] addressed the brain mechanisms of the sense of self-ownership and showed that a threshold of detectable visual feedback delay of one's own body movement was about 200 ms. Remarkably, the temporal discrepancy between visual and proprioceptive feedbacks modulated the activity in the parietal areas, which is considered to be involved in the processing of one's own body and those of others. Modulation of parietal activity as a function of the synchronous and asynchronous conditions in an RHI experiment has also been reported in previous studies [7], [8]. Based on these reports, we hypothesized that temporal discrepancy in the range of 200–500 ms between visual and tactile stimulations would be sufficient to attenuate RHI. In order to examine this hypothesis, we conducted a behavioral RHI experiment in which a temporal discrepancy, ranging from 100–600 ms at 100 ms intervals, between touching the subject's own hand and touching the rubber hand was systematically introduced by using a visual feedback delay experimental set-up.

## Materials and Methods

### Participants

Eighteen healthy male undergraduate students, who were naïve as to the purpose of the study, were recruited for the experiments (age 22.2±0.5 years, mean±SD), on the basis of written informed consent. Another group of six subjects participated in this study but were excluded because they reported drowsiness during the experiment and showed little RHI effect. All subjects were right-handed and had normal or corrected-to-normal vision. The experiments were approved by the ethics committee of School of Science and Technology, Meiji University, and conducted according to the principles and guidelines of the Declaration of Helsinki.

### Experimental Setup and Methods

**Figure 1:**
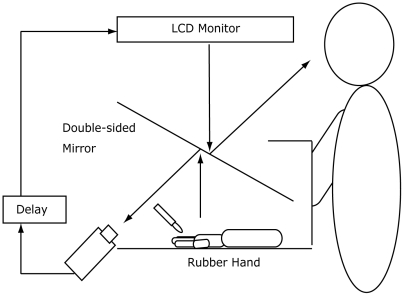
The experimental set-up. The subject watched a delayed image of the rubber hand that was touched synchronously with the subject's own hand. The length of the visual feedback delay ranged from 100 to 600 ms at 100 ms intervals.

The subjects were asked to sit at a table and put their right hand on the table with its palm facing down. A life-sized right rubber hand rested on the table 15 cm on the left from the subject's right hand. A double-sided tilted mirror was installed above the table, so that the subjects were not able to directly see the rubber hand or their own right hand (Fig. 1). The image of the rubber hand that was reflected in the back side of the mirror was filmed using a video camera (HDR-HC3, SONY, Tokyo, Japan). The rubber hand image was presented on a liquid-crystal monitor (LMD-232W, SONY, Tokyo, Japan) set above the mirror, and the subject could see the reflected image of the rubber hand in the front side of the mirror. The angle of the mirror was finely adjusted before the experiment so that the rubber hand was viewed from the subject as if it was placed horizontally on the table.

Visual feedback delay was introduced using a hardware device (EDS3305, ELETEX, Osaka, Japan) connected between the video camera and the monitor. Six delay conditions ranging from 0 to 500 ms at 100 ms intervals were tested. The intrinsic delay of the visual feedback in this experimental setting was approximately 85 ms as measured by a high-speed camera (OPAL-1000, Adimec, Eindhoven, Netherlands). Thus the delay conditions effectively ranged from 85–585 ms. For simplicity, we refer to these delays as 100–600 ms hereafter.

Subjects were instructed to fixate on the rubber hand throughout the 3 min stimulation period, during which the index finger of the subject's own right hand and that of the rubber hand were stroked as simultaneously as possible using two paintbrushes that were connected tightly with each other in a U-shape. The experimenter touched both hands in an unpredictable manner at approximately 0.5–1 Hz. Although both the subject's hand and the rubber hand were always stimulated synchronously, the subject saw a delayed image of the rubber hand (varied from 100–600 ms) because of our experimental settings described above. The length of the delay was constant throughout each stimulation period. Because the same well-trained experimenter conducted all experiments, we could assume that possible temporal fluctuations between the concurrent two brushstrokes were minimal and did not vary across the delay conditions or across subjects. Six delay conditions were tested for each subject with 5-minute inter-trial breaks. The order of the delay conditions was pseudo-random and counterbalanced across subjects.

Immediately before and after the stimulation period, the subjects were required to estimate the position of their own right index finger; the subject reached with their left index finger from below the table to the estimated position of their right index finger. The proprioceptive drift was defined as the lateral difference in the reached positions before and after the stimulation period. After each trial, subjects completed a Japanese translated version of a questionnaire identical to that used in a previous study [1]. First three items were regarded as indicators of occurrence of RHI, while the remaining items (4–9) served as control (see Appendix S1). A 7-pointed visual-analog scale ranging from −3 (strongly disagree) to +3 (strongly agree) was used. To analyze the effect of the length of visual feedback delay, a linear regression analysis as well as one-way repeated measures analysis of variance (ANOVA) were applied to the scores on the questionnaire items. The data were also submitted to t-tests with false discovery rate (FDR) control [10] to examine whether there was a significant RHI effect (higher than zero). The significance level for all statistical tests was set at 0.05.

## Results

**Figure 2:**
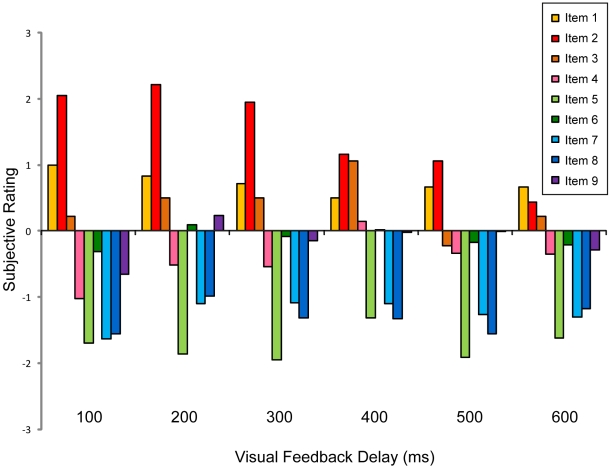
Subjective rating on questionnaire item 1–9 as a function of visual feedback delay length. First three items (1–3) were regarded as indicators of occurrence of RHI.

**Figure 3:**
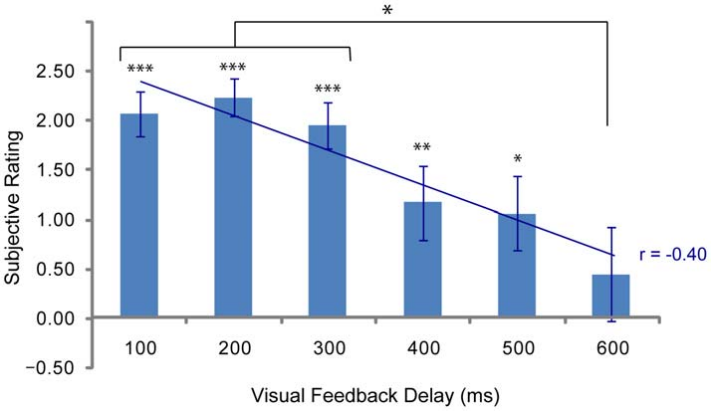
Subjective rating on questionnaire item 2. There were significant RHI effects in the 100 thrhough 500 ms delay conditions (***: P<0.001, **: P<0.01, *: P<0.05, corrected). There were significant differences between 100–300 and 600 ms conditions (P<0.05; Tukey HSD test).

Fig. 2 depicts subjective rating results on questionnaire items as a function of visual feedback delay length. Linear regression analyses showed a significant correlation of scores on item 2, which is an indicator of the occurrence of RHI, with visual feedback delay length (r = −0.401, N = 108, P<0.001; Fig. 3), but not of other items (P>0.1). Scores on item 2 showed the illusionary effects in the minimum (100 ms) delay condition (score  = 2.06±0.94, t(17) = 9.30, P<0.001, corrected) and became smaller as visual feedback delay length became longer. ANOVA revealed that a main effect of visual feedback delay was observed for item 2 (F(5, 85) = 7.81, P<0.01). Item 1 showed nearly significant illusionary effects in the minimum delay condition (score = 1.00±1.57, t(17) = 2.70, P = 0.06, corrected; P<0.01, uncorrected), but failed to show differences between conditions (F(5, 85) = 0.37, P>0.1). No significant difference between conditions was observed for any other item (P>0.1, ANOVA). Thus further analyses were performed only on item 2. Significant RHI effects in the 100 through 500 ms delay conditions were observed (P<0.05, corrected; Fig. 3). Subsequent post-hoc analyses revealed that there were significant differences between the 100–300 ms conditions and the 600 ms condition (P<0.05; Tukey HSD test; Fig. 3).

The anlayses on proprioceptive drift using t-tests with FDR control showed similar results, although significant differences among conditions were not observed by means of regression analyses (r = –0.08, p>0.1) or ANOVA (F(5, 85) = 1.00, P>0.1). There were significant proprioceptive drifts in the 200 and 300 ms delay conditions (t(17) = 3.22 and t(17) = 3.15, respectively, P<0.05, corrected; Table 1), and a marginal drift was observed in the 100 ms delay condition (t(17) = 2.04; P = 0.08, corrected). No significant proprioceptive drifts were observed in longer conditions (P>0.1 in the 400 and 600 ms conditions, P = 0.09 in the 500 ms condition).

**Table 1:** Proprioceptive drift in each delay condition.

Delay (ms)	Mean (cm)	SD (cm)	t-value
100	1.10	2.28	2.04† (P = 0.08)
200	1.50	1.98	3.22*
300	1.34	1.81	3.15*
400	0.43	2.11	0.86
500	1.33	3.18	1.77† (P = 0.09)
600	0.59	2.27	1.10

* p<0.05.

† P<0.1.

## Discussion

Our results demonstrated that temporal contiguity between visual and tactile stimulations is needed to induce RHI. Remarkably, the RHI effect in terms of subjective rating became smaller with increasing visual feedback delay length, and the effect was significantly stronger in less than 300 ms delay conditions than in 600 ms delay conditions. Weaker, but still significant, RHI effects were observed in 400 and 500 ms delay conditions, but not in 600 ms. Results for proprioceptive drifts showed similar RHI effects. Our results not only support the hypothesis that RHI is greatly attenuated with temporal discrepancies of more than 500 ms between visual and tactile stimulations, but also revealed that a temporal discrepancy of less than 300 ms between the two stimulations is preferable to induce a strong sensation of RHI.

Previous studies on multi-sensory integration regarding self-body have shown that a temporal discrepancy of 200–300 ms between different sensory modalities induces conscious detection of the multi-sensory discrepancy [9], [11]. In our previous study, subjects were presented with delayed visual feedback of involuntarily moving their own hand and could detect the visual feedback delay if the temporal discrepancy between the visual and proprioceptive feedback exceeded 200 ms [9]. Leube et al. [12] showed similar results when the subject saw the delayed visual feedback of a self-generated hand action. These studies are similar yet differ in the sense that the former study addressed a passive movement of one's own hand while the latter addressed an active movement. Interestingly, Blakemore et al. [13] reported that the subject felt ticklishness when a tactile sensation delayed by more than 300 ms from a self-generated action was given to the other hand, while shorter delayed tactile sensation caused little ticklishness. These results suggest that stimuli that are delayed by more than 300 ms are not processed as self-generated. These results concur with the view that the brain requires temporal contiguity of 200–300 ms to integrate visual and tactile/proprioceptive inputs (feedbacks) for self-body processing. The present study is consistent with these studies in that a discrepancy of less than 300 ms between the visual and tactile stimuli was required for the marked amplitude of RHI. It is worth noting that we did not examine RHI effect in 0 ms delay condition due to limitation of our experimental set-up. However, the result in the synchronous condition in the previous study [1] (score is approximately 2.3 for item 2, P<0.018, corrected) seems not to be different from our result in the minimum (100 ms) delay condition (score = 2.06, P<0.001, corrected).

In our experiment, a proprioceptive drift was observed for the shorter delay conditions (≤300 ms), while no significant drift was observed in the longer delay conditions, although we failed to find a conditional difference (P>0.1, ANOVA). Less robust results of proprioceptive drift in our experiments may be partially due to the experimental set-up, where subjects saw the rubber hand reflected in the mirror. Although we carefully set up the mirror to reflect the monitor image of the rubber hand as if it was placed horizontally on the table, subjects might have perceived the spatial incongruity of the hand and/or lack of 3-D information that might have caused the deviation in the pointing movement. Recently, Kammers et al. [14] reported that the perceptual judgment in RHI was more sensitive than the proprioceptive drift. Less robust conditional effects on the proprioceptive drift in our experiments were in line with their findings. Subjective rating results on item 3 in our study were also less sensitive as compared to previous studies [1]. Considering one RHI study with Japanese subjects that reported little RHI effect on this questionnaire item [15], the insensitivity to this item may be due to a cultural difference, although further study is needed to clarify this point.

RHI indicated that the visual image of a hand-like object concordant with a tactile sensation is attributed to self-body. Our results showed that the required time window for this vision-tactile concordance is less than 500 ms, and that, a time window of less than 300 ms is preferable for achieving a strong sensation of self-body attribution. This result is close to the time window of approximately 200 ms that the subject needed to judge body movement as their own [9], [11]. We suggest that the temporal contiguity required to induce RHI is closely related to the subjective feeling of temporal consistency among sensory inputs, which is likely processed in the parietal cortex [8], [9]. The mechanism underlying RHI that attributes a visual object to the self likely overlaps with the mechanism that detects concordance between visual and tactile/proprioceptive feedbacks. A visual object that subjectively coincides with tactile/proprioceptive sensation would be flexibly incorporated into the internal self-body representation.

## Supporting Information

Appendix S1 Questionnaire items.
(0.02 MB DOC)

## References

[1] Botvinick M, Cohen J (1998). Rubber hands ‘feel’ touch that eyes see.. Nature.

[2] Armel KC, Ramachandran VS (2003). Projecting sensations to external objects: evidence from skin conductance response.. Proc R Soc Lond B - Biol Sci.

[3] Costantini M, Haggard P (2007). The rubber hand illusion: sensitivity and reference frame for body ownership.. Conscious Cogn.

[4] Haans A, Ijsselsteijn WA, de Kort YA (2008). The effect of similarities in skin texture and hand shape on perceived ownership of a fake limb.. Body Image.

[5] Press C, Heyes C, Haggard P, Eimer M (2008). Visuotactile learning and body representation: an ERP study with rubber hands and rubber objects.. J Cogn Neurosci.

[6] Makin TR, Holmes NP, Ehrsson HH (2008). On the other hand: dummy hands and peripersonal space.. Behav Brain Res.

[7] Ehrsson HH, Spence C, Passingham RE (2004). That's my hand! Activity in premotor cortex reflects feeling of ownership of a limb.. Science.

[8] Tsakiris M, Hesse MD, Boy C, Haggard P, Fink GR (2007). Neural signatures of body ownership: a sensory network for bodily self-consciousness.. Cereb Cortex.

[9] Shimada S, Hiraki K, Oda I (2005). The parietal role in the sense of self-ownership with temporal discrepancy between visual and proprioceptive feedbacks.. NeuroImage.

[10] Benjamini Y, Hochberg Y (1995). Controlling the false discovery rate: a practical and powerful approach to multiple testing.. J R Statist Soc Ser B.

[11] Franck N, Farrer C, Georgieff N, Marie-Cardine M, Dalery J (2001). Defective recognition of one's own actions in patients with schizophrenia.. Am J Psychiatry.

[12] Leube DT, Knoblich G, Erb M, Grodd W, Bartels M (2003). The neural correlates of perceiving one's own movements.. NeuroImage.

[13] Blakemore SJ, Frith CD, Wolpert DM (1999). Spatio-temporal prediction modulates the perception of self-produced stimuli.. J Cogn Neurosci.

[14] Kammers MP, de Vignemont F, Verhagen L, Dijkerman HC (2009). The rubber hand illusion in action.. Neuropsychologia.

[15] Kanayama N, Sato A, Ohira H (2007). Crossmodal effect with rubber hand illusion and gamma-band activity.. Psychophysiology.

