# Supplementary material for: Rubber Hand Illusion under Delayed Visual Feedback
Source: PLoS One. 2009 Jul 9;4(7):e6185. doi: 10.1371/journal.pone.0006185 (PMC2702687; doi:10.1371/journal.pone.0006185)
Supplement: Appendix S1 — Questionnaire items. (0.02 MB DOC) [file pone.0006185.s001.doc]

## Appendix S1

Questionnaire items:

1) It seemed as if I were feeling the touch in the location where I saw the rubber hand being touched.

2) It seemed as though the touch I felt was caused by the paintbrush touching the rubber hand.

3) I felt as if the rubber hand were my hand.

4) I felt as if my (real) hand were drifting towards the left (towards the rubber hand).

5) It seemed as if I might have more than one right hand or arm.

6) It seemed as if the touch I was feeling came from somewhere between my own hand and the rubber hand.

7) It felt as if my (real) hand were turning ‘rubbery’.

8) It appeared (visually) as if the rubber hand were drifting towards the right (towards my hand).

9) The rubber hand began to resemble my own (real) hand, in terms of shape, skin tone, freckles or some other visual feature.
